# Phonological Variations Are Compensated at the Lexical Level: Evidence From Auditory Neural Activity

**DOI:** 10.3389/fnhum.2021.622904

**Published:** 2021-04-27

**Authors:** Hatice Zora, Tomas Riad, Sari Ylinen, Valéria Csépe

**Affiliations:** ^1^Department of Linguistics, Stockholm University, Stockholm, Sweden; ^2^Department of Swedish Language and Multilingualism, Stockholm University, Stockholm, Sweden; ^3^CICERO Learning, Faculty of Educational Sciences, University of Helsinki, Helsinki, Finland; ^4^Cognitive Brain Research Unit, Faculty of Medicine, University of Helsinki, Helsinki, Finland; ^5^Brain Imaging Centre, Research Centre for Natural Sciences, Budapest, Hungary

**Keywords:** phonology, assimilation, lexical access, MMN, Swedish

## Abstract

Dealing with phonological variations is important for speech processing. This article addresses whether phonological variations introduced by assimilatory processes are compensated for at the pre-lexical or lexical level, and whether the nature of variation and the phonological context influence this process. To this end, Swedish nasal regressive place assimilation was investigated using the mismatch negativity (MMN) component. In nasal regressive assimilation, the coronal nasal assimilates to the place of articulation of a following segment, most clearly with a velar or labial place of articulation, as in *utan mej* “without me” > [ʉːtam mɛjː]. In a passive auditory oddball paradigm, 15 Swedish speakers were presented with Swedish phrases with attested and unattested phonological variations and contexts for nasal assimilation. Attested variations – a coronal-to-labial change as in *utan* “without” > [ʉːtam] – were contrasted with unattested variations – a labial-to-coronal change as in *utom* “except” > ^∗^[ʉːtɔn] – in appropriate and inappropriate contexts created by *mej* “me” [mɛjː] and *dej* “you” [dɛjː]. Given that the MMN amplitude depends on the degree of variation between two stimuli, the MMN responses were expected to indicate to what extent the distance between variants was tolerated by the perceptual system. Since the MMN response reflects not only low-level acoustic processing but also higher-level linguistic processes, the results were predicted to indicate whether listeners process assimilation at the pre-lexical and lexical levels. The results indicated no significant interactions across variations, suggesting that variations in phonological forms do not incur any cost in lexical retrieval; hence such variation is compensated for at the lexical level. However, since the MMN response reached significance only for a labial-to-coronal change in a labial context and for a coronal-to-labial change in a coronal context, the compensation might have been influenced by the nature of variation and the phonological context. It is therefore concluded that while assimilation is compensated for at the lexical level, there is also some influence from pre-lexical processing. The present results reveal not only signal-based perception of phonological units, but also higher-level lexical processing, and are thus able to reconcile the bottom-up and top-down models of speech processing.

## Introduction

Lexical access, the matching of auditory input onto lexical representations in the brain, is an essential component of speech perception. Although seemingly simple and effortless, it is a complex process given that speech is inherently highly variable. Changes in phonological shapes due to various factors, such as speech rate, dialect, coarticulation, and assimilation, make each pronunciation unique. Assimilation, which is the focus of the present paper, changes the surface forms of spoken words. It occurs when a sound is influenced by a neighboring segment and accommodates some aspect of it, as in the following Swedish example: *en båt* “a boat” > [ɛm boːt] where assimilation concerns the place of articulation of /n/. Although making articulation easier, this process introduces variability that the perceptual system has to deal with as phonological contrasts are neutralized and the lexical form of an item becomes less directly reflected. Several theories have been suggested to explain the processing of assimilatory variations, however, neither the nature of these variations and their consequences in lexical access nor the neural correlates of auditory matching mechanisms in this process are fully understood.

The aim of the present research is to investigate how listeners deal with attested phonological assimilations and unattested phonological variations during lexical access, and to elaborate on the findings with regard to previous theoretical accounts ranging from (i) *simple lexical compensation* accounts to (ii) *feature underspecification*, (iii) *regressive inference*, and (iv) *feature parsing* accounts (for an overview, see Gow, 2001; Jusczyk and Luce, 2002; Ranbom and Connine, 2007; Gaskell and Snoeren, 2008; Darcy et al., 2009; Lahiri and Reetz, 2010). Deriving from the different assumptions of these accounts, the objectives are to assess the auditory assimilatory processes operating at the pre-lexical or lexical level, the role of contextual information justifying the assimilation, and the nature of information required for the auditory matching, being either discrete phonological features, or gradient phonetic details for the perception of assimilation. These objectives are achieved by scrutinizing the neural correlates of this near-instantaneous perceptual process using the mismatch negativity (MMN) component of auditory event-related potentials (ERPs) in a well-balanced paradigm enabling the comparison of different theoretical assumptions. The MMN is considered an optimal tool to investigate the attested and unattested phonological variations given that it not only reflects the auditory variations but also the linguistic relevance of these variations in early speech comprehension processes. In the following, we first give an overview of four different theoretical accounts for phonological assimilation, present MMN studies investigating assimilatory processes, and then formulate MMN predictions based on these theoretical accounts.

The so-called *lexical compensation* account relies on stored lexical information to explain the spontaneous lexical access despite the changes in the surface forms. The auditory input is matched with words stored in the mental lexicon and the best-matching lexical item is retrieved among multiple candidates. In this account, candidates may be activated by incomplete input. A minimal mismatch between the features that are extracted from the signal and the features that comprise the lexical representation is compensated for by the listeners, and thus *gree*[m] might successfully activate *green* and any similar sounding words, since there is only one contradictory feature in the input (Marslen-Wilson and Welsh, 1978; Connine et al., 1993; Bölte and Coenen, 2002). Changes in the phonological shape and phonetic variations are tolerated based on higher-order top-down information such as semantic and syntactic contexts (Marslen-Wilson and Welsh, 1978; Samuel, 2001; Bölte and Coenen, 2002; Darcy et al., 2009; see also the TRACE model of McClelland and Elman, 1986).

Some researchers argue that the tolerance for phonological variation depends on the specification of features in the mental lexicon, such that only variations that do not mismatch the features specified in the lexical entry are allowed without obstruction of lexical access. According to the *featurally underspecified lexicon account* (FUL; Lahiri and Marslen-Wilson, 1991; Lahiri and Reetz, 2002, 2010), for instance, the acoustic features extracted from the speech signal are matched with the phonological features in the lexicon, which stores only specified features constrained by language-specific properties. Features that exhibit variation based on the segmental or prosodic context and that can therefore be assigned by rule are not retained in the lexicon. Rather, such features are considered predictable and underspecified. The features *labial* and *dorsal*, for instance, are represented in the mental lexicon, while the feature *coronal* is considered underspecified. The coronal feature, as the universal place feature, can be activated not only by coronal but also by non-coronal information. Coronal phonemes are thus more likely to assimilate to non-coronal phonemes than the other way around. For example, labial [m] activates lexical representations of both /m/, which is specified for a labial place of articulation, and coronal /n/, which is underspecified for the place of articulation. Accordingly, *gree*[m] in a labial context as in *bean* will activate the word *green*. However, *sa*[n]*e* in a coronal context as in *duck* will not activate the word *same*. Based on these feature specifications, the FUL account suggested a ternary matching condition for the activation of word candidates: *match*, *mismatch*, and *no-mismatch*. Depending on the same or contradictory features between the signal and the representations in the lexicon, a *match* or a *mismatch* occurs, respectively. While a *match* accelerates the activation of potential candidates, a *mismatch* eliminates words as candidates. Full match and mismatch of features are not, however, the only alternatives since the lexicon would tolerate surface variations if features do not conflict. A *no-mismatch* reflects instances where (i) no feature, which is part of the mental lexicon, is extracted from the signal, or (ii) a feature, which is underspecified for the place of articulation, is extracted from the signal. A no-mismatch condition neither excludes candidates nor precludes lexical access, but receives less activation than a perfect match. Word candidates are activated according to the number of matching features as specified in the mental lexicon and the number of features extracted from the signal, along with the higher-order information (Lahiri and Reetz, 2002, 2010; Felder, 2009).

According to the FUL account, phonological underspecification is insensitive to assimilatory processes and phonological context. The *gree*[m] example above will thus activate the word *green* regardless of the following context. Experimental evidence for the indifference to the assimilatory processes and the phonological context has been indicated in a number of priming studies (Lahiri, 1995; Lahiri and van Coillie, 1999; Lahiri and Reetz, 2002; Wheeldon and Waksler, 2004). For instance, Lahiri and van Coillie (1999; cited in Lahiri and Reetz, 2002) indicated that *Bah*[m] (*Bahn* “railway” in a labial context) presented in isolation primed the semantically related word *Zug* “train” as much as the word *Bahn* did, in comparison to the unrelated word *Maus* “mouse,” whereas *Lär*[n] (*Lärm* “noise” in a coronal context) did not prime the semantically related word *Krach* “bang” as much as the word *Lärm* did. There is, however, some research providing evidence for the role of phonological context in assimilatory processes (Gaskell and Marslen-Wilson, 1996, 2001; Coenen et al., 2001; Mitterer and Blomert, 2003; Mitterer et al., 2006; Gaskell and Snoeren, 2008). Using cross-modal priming, Gaskell and Marslen-Wilson (1996) for instance suggested that the perceptual system is more tolerant of assimilatory changes where the place of articulation of the following context matches with the place of articulation of the assimilated segment. According to this so-called *regressive inference account*, the perceptual system is faster and more accurate in processing assimilatory changes in phonologically appropriate contexts as in the Swedish example *en båt* “a boat” [ɛm boːt], above, than in inappropriate contexts as in ^∗^[ɛm toː] for *en tå* “a toe.”

Another account that argues for context sensitivity is called the *feature parsing account* (Gow, 2001, 2003). The feature parsing account is based on acoustic processes that hold across languages, and covers both coarticulation and phonological assimilation. This account bases assimilatory operations on a pre-lexical level through basic perceptual grouping principles, and argues that the assimilated segment carries information not only about the original place of articulation present in the signal but also about the following segment (Nolan, 1992; Gow, 2000). Given the Swedish example above, [m] of the altered [ɛm] carries not only the properties of labiality of [b] of *båt*, but also the original properties of /n/ of *en*. If no trace of coronality is left in [ɛm], the original *en* cannot be parsed. Similarly, the bilabial cues cannot be parsed in the absence of a following bilabial consonant as in ^∗^[ɛm to ː]. In this account, some mismatches between the extracted and expected features are tolerated, and this tolerance does not rely on the phonological nature of the variation causing the mismatch (cf. the FUL account above). Gow (2001), for instance, compared a phonological assimilation such as *gree*[m] *boat* with an example of an unattested phonological variation as in *^∗^glu*[n] *day* in a priming study. According to the FUL account, while *gree*[m] will prime *green* (a no-mismatch condition), the unattested variation *^∗^glu*[n] should not prime *glum* (a mismatch condition). The findings in Gow (2001), however, indicated no difference in priming for the two conditions. In another study, Gow (2003) investigated how listeners process assimilations by examining ambigious segments covering the acoustic properties of both coronals and labials (e.g., *cone* pronounced as [kon/m]). The results indicated, among other things, that the listeners accessed the labial alternative *comb* when the next segment was coronal as in *dents*. This was explained through perceptual grouping principles, which predict that the coronality of the /n/ in *cone* should group with the coronality of the /d/ in *dents*, and thus the coronality would be removed from the assimilated segment, which in turn would leave only the labial property to be associated with the final segment in [kon/m]. Coarticulated features in the assimilated segment are associated with the following assimilation context, and residuals of coarticulation are used to predict an upcoming segment.

Given that the matching of auditory input onto representations in the brain, be they lexical, phonological or acoustic, is near-instantaneous, the distinct neurophysiological patterns of these theoretical accounts can ideally be examined with the MMN component of ERPs, which can reflect the brain’s automatic auditory information processing as early as 150–250 ms from stimulus onset. The MMN response is typically investigated using a passive oddball paradigm, where a rare stimulus (deviant) is interspersed among frequent stimuli (standard), and is elicited even when attention is directed elsewhere (Näätänen et al., 1978, 2007; Paavilainen et al., 2007; Winkler, 2007). The MMN response is optimal for investigating assimilatory processes at the pre-lexical and lexical levels, as it reflects not only low-level acoustic processing but also higher-level cognitive and linguistic processes such as activation and formation of long-term memory representations and predictive processes (Pulvermüller et al., 2001; Ylinen et al., 2010, 2016; Zora et al., 2015, 2016a, b, 2019; Garami et al., 2017). Given that the amplitude of MMN depends on the degree of variance between the stimuli (Sams et al., 1985; Pakarinen et al., 2007), several studies used the MMN component to investigate the variations introduced by assimilatory processes and their consequences for the auditory neural activity (Mitterer and Blomert, 2003; Mitterer et al., 2006; Tavabi et al., 2009). Some of these studies are reviewed in detail below.

Mitterer and Blomert (2003) investigated the phonological context dependency of assimilation in Dutch, and examined an attested change (from coronal /n/ to labial /m/) in appropriate and inappropriate phonological contexts as in *tuinbank* “garden bench” [tɶynbɑŋk] and *tuinstoel* “garden chair” [tɶynstu

l], respectively. The authors hypothesized that if there is a regressive inference mechanism, the perceptual distance between *tuinbank* [tɶynbɑŋk] and [tɶymbɑŋk] (i.e., appropriate context) should be smaller than the distance between *tuinstoel* [tɶynstu

l] and ^∗^[tɶymstu

l] (i.e., inappropriate context), and accordingly, the MMN should be smaller in the appropriate context than in the inappropriate context. In line with these expectations, the results indicated smaller MMN to the assimilation of /n/ to [m] in the appropriate context [tɶymbɑŋk] than in the inappropriate one ^∗^[tɶymstu

l]. The authors thus argued that an [m] that is followed by a [b] is perceived as a version of /n/ in automatic auditory processing, and concluded that phonological assimilations are coped with by early pre-lexical mechanisms rather than by lexical top-down mechanisms.

The MMN component has also been used to investigate whether the processing of phonological assimilations is affected by language experience and phonetic details of assimilated segments. In a series of experiments, Mitterer et al. (2006) examined Dutch listeners’ perception of Hungarian liquid assimilation (from /l/ to [r]) as compared to that of native Hungarian participants. In the first experiment, MMN responses to Dutch words, where Hungarian liquid assimilation was applied as in [knɑlroːt] “vivid red” > [knɑrroːt], were recorded. As a control, an unattested variation as in [knɑlblɑu] “vivid blue” > ^∗^[knɑrblɑu] was used. Similar to the findings of Mitterer and Blomert (2003), the MMN elicited by [knɑrroːt] was smaller than the MMN elicited by ^∗^[knɑrblau]. The authors argued that Dutch listeners handle Hungarian liquid assimilation similarly to Dutch nasal place assimilations, and claimed therefore that processing of assimilations does not rely on language experience.

In the second experiment, Mitterer et al. (2006), tested whether Hungarian listeners process their native liquid assimilation in a context-dependent way like the Dutch listeners. To this end, MMN responses to Hungarian words with Hungarian liquid assimilation as in [bɔlroːl] > [bɔrroːl] and [bɔlnaːl] > ^∗^[bɔrnaːl] were investigated. Since, according to the liquid assimilation rule in Hungarian, the change from /l/ to /r/ is expected before the delative suffix [roːl] but not before the adessive suffix [naːl]^1^, the authors predicted the MMN to [bɔrroːl] to be smaller than the MMN to ^∗^[bɔrnaːl]. However, in contrast to the context sensitivity documented with Dutch listeners in the first experiment, the MMNs did not differ between these conditions. To see if the language background of the listeners might explain this difference, in the third experiment, the authors presented the stimuli used in the second experiment to Dutch listeners. These results were, however, similar to the ones obtained with Hungarian listeners, and the authors concluded that the difference in language background could not explain the different results between the first and second experiments.

Mitterer et al. (2006), in the fourth experiment, examined whether the acoustic quality of the stimuli were responsible for the difference in results. To this end, the authors presented altered versions of the Hungarian words (with a comparably weak /r/) used in the second and third experiments to Dutch listeners. Similar to the first experiment, the results indicated significant MMN for the inappropriate context, but not for the appropriate context, and accordingly the authors argued that context-sensitive MMN elicitation depends on the acoustic details of the stimuli. In the fifth experiment, the authors repeated the fourth experiment with Hungarian participants, and the results replicated the findings in the first and fourth experiment. The authors concluded that assimilatory processes do not rely on previous experience with a given assimilation rule. However, the phonetic details of the assimilated segment affect this process; assimilations are tolerated only when the assimilated phoneme is a weak example of the category. The authors argued that assimilatory processes take place at a pre-lexical level, independently of specific language experience in a similar fashion to coarticulatory compensation. The authors further claimed that as articulatory simplifications, assimilations are constrained by perception, and general perceptual preferences have an impact on the kind of assimilation rules applied.

This pre-lexical processing mechanism for assimilations has also been indicated using pseudowords in Tavabi et al. (2009). The authors investigated both the frequency of variation and the contextual appropriateness in nasal regressive place assimilation in items with no lexical representation. Frequent changes (from /n/ to /m/) were contrasted with rare changes (from/m/to/n/) in appropriate and inappropriate contexts (/b/or/d/). The MMN responses indicated an asymmetry in neural activity between the frequent and rare changes only in the appropriate context condition. While the rare changes elicited a much larger MMN response than the frequent changes in the appropriate contexts, the frequency of change had no significant effect in the inappropriate contexts. The authors argued that since the results were obtained using pseudowords, the lexical level is not essential for assimilatory processes in line with previous findings (Mitterer et al., 2006). The authors argued that although the results on the frequency of change provide some evidence for the FUL account, given the observed interaction between the frequency of change and the context appropriateness, their results are better understood with the feature parsing and inference accounts, which also argue for assimilatory processes operating on a pre-lexical level.

As noted earlier, the present paper aims to investigate the consequences of attested phonological assimilation and unattested phonological variation in lexical access, and to parse out neural correlates of their potential effects at the pre-lexical or lexical level using the MMN component. The processing strategies (if any) will also be elaborated in light of previous accounts as presented above. To this end, phonological variation introduced by Swedish nasal regressive place assimilation was compared to an instance of unattested phonological variation that does not appear naturally in the language. In Swedish, as in many other languages, the coronal nasal assimilates to the place of articulation of a following segment as in *en morgon* “a morning” [ɛm mɔrːɡɔn], whereas the labial nasal stays unaffected (e.g., *fem nålar* “five needles” [fɛm noːlar] > ^∗^[fɛn noːlar])^2^ (Riad, 2014). In a well-balanced paradigm, we investigated an attested variation introduced by nasal regressive place assimilation (i.e., from coronal/n/to labial [m]) in an appropriate context as in [ʉːtan mɛjː] “without me” > [ʉːtam mɛjː] as well as in an inappropriate context [ʉːtan dɛjː] “without you” > ^∗^[ʉːtam dɛjː]. In addition and for comparison, the context sensitive interpretation of an unattested change (i.e., from labial/m/to coronal [n]) as in [ʉːtɔm mɛjː] “except me” > ^∗^[ʉːtɔn mɛjː] and [ʉːtɔm dɛjː] “except you” > ^∗^[ʉːtɔn dɛjː] was investigated. These phrases were presented in four oddball blocks; unaltered canonical versions of the phrases always served as standards ([ʉːtan mɛjː], [ʉːtan dɛjː], [ʉːtɔm mɛjː], [ʉːtɔm dɛjː]), and altered versions, whether with expected assimilation or not, served as deviants ([ʉːtam mɛjː], ^∗^[ʉːtam dɛjː], ^∗^[ʉːtɔn mɛjː], and ^∗^[ʉːtɔn dɛjː]). By examining the nature of variation and the phonological context in assimilatory processes using real words, the present paper introduces an improvement on the methodology of earlier MMN studies on the topic (Mitterer and Blomert, 2003; Mitterer et al., 2006; Tavabi et al., 2009). Although using real words, Mitterer and Blomert (2003) and Mitterer et al. (2006) looked at contextual appropriateness but did not fully investigate the nature of the change and lacked a control condition for an unattested phonological variation. Tavabi et al. (2009), although investigating both contextual appropriateness and the nature of the change, used only pseudowords and, therefore, focused only on the pre-lexical level. The current experimental paradigm admits the evaluation and comparison of different theoretical accounts. Given the sensitivity of MMN responses to any auditory differences (be it sensory or cognitive), the current experimental stimuli, which consist of phonetically and functionally identical words, critically allow the comparison of attested and unattested variations on equal grounds to a large extent in a diagonal design.

The theoretical accounts presented above are not fully exclusive. However, they differ in ascribing different roles to (i) the processing stage (pre-lexical vs. lexical), (ii) the relevance of contextual information, and (iii) the nature of information required for auditory matching (discrete phonological features vs. gradient phonetic details) for the perception of assimilation. The simple lexical compensation and FUL accounts both implement at the lexical level, and are insensitive to phonological context. But they differ in the representation of features in the mental lexicon. The former holds that all features of a word are fully specified and represented in the mental lexicon. According to this account, the perceptual system treats variations in the speech input as random noise and the higher-order information is employed to recover the signal from noise. The FUL account, on the other hand, argues that only specified features are stored in the mental lexicon, and words are activated depending on the number of matching features as specified in the lexical entries and the number of features extracted from the signal, along with the higher-order information. Both the regressive inference and feature parsing accounts claim for a pre-lexical processing stage for assimilations, and assert that the contextual appropriateness is crucial for the assimilatory processes, in contrast to the claims of simple lexical compensation and FUL accounts. However, while the regressive inference account relies on phonological rules and constraints, the feature parsing account builds on the gradient phonetic details in the signal and the language independent acoustic processes. In the feature parsing account, the auditory matching procedure does not rely on the specification of features in the mental lexicon as argued in the FUL account.

Depending on these differences across the major theoretical accounts, different patterns of MMN responses are predicted across experimental blocks (see Table 2). According to the simple lexical compensation account, there should not be any difference across these blocks since the correct forms would be retrieved, irrespective of the nature of the variation and the following phonological context, using semantic context. Thus, only acoustic MMN responses would be predicted in any of these blocks since assimilations would be compensated for at the lexical level. According to the FUL account, on the other hand, MMN responses are predicted to differ across [ʉːtan] and [ʉːtɔm], yet irrespective of the following phonological contexts, [mɛjː] and [dɛjː]. Accordingly, in both Block I and II, the deviants should be tolerated given the assimilation of [n] in [ʉːtan] to [m] due to the underspecification of coronal /n/ (no-mismatch condition), and consequently a smaller MMN response is predicted to the deviants. In Blocks III and IV, on the other hand, the deviants should not be tolerated by assimilation of the [m] in [ʉːtɔm] to [n], since nasal assimilation only applies to the coronal nasal (mismatch condition), and consequently a clear MMN response is predicted to the deviants. In contrast to the FUL account, according to the regressive inferential account, MMN responses are predicted to differ across [ʉːtan] and [ʉːtɔm] depending on the following context, [mɛjː] and [dɛjː]. In Block I, the deviant should be tolerated by assimilation of the [n] in [ʉːtan] to [m] due to the following phonological context [mɛjː], and accordingly no MMN response is predicted to the deviant. In Block II, on the other hand, the deviant should not arise by assimilation of the /n/ in [ʉːtan] to [m], due to the lack of an appropriate following context for assimilation, and an MMN response is predicted to be elicited to the deviant. No direct MMN responses can be predicted according to the feature parsing account, given that the assimilated segments in the present paper consist of unmodified coronals and labials rather than segments which are phonetically ambiguous and show acoustic characteristics intermediate between underlying and surface forms, as tested in the feature parsing account. However, given that the labiality of the/m/[ʉːtam] should group with the labiality of the /m/ in [mɛjː], leaving only the coronal property to be associated with the final segment of the preceding word as in [ʉːtan], and the coronality of the /n/ in [ʉːtɔn] should group with the coronality of the /d/ in [dɛjː], thus leaving only the labial property to be associated with the final segment^3^ in [ʉːtɔm], an attenuated MMN response is predicted to the deviants in Blocks I and IV compared to the deviants in Blocks II and III.

## Materials and Methods

### Participants

The participants were 15 native speakers of Swedish (8 females, 7 males; age range 19–37 years, *M* = 28.06, and *SD* = 5.13). All participants were strongly right-handed as assessed with the Edinburgh Handedness Inventory (Oldfield, 1971) and reported normal development and hearing.

### Ethics Statement

Written informed consent was obtained from all participants before testing. The study complied with the ethical guidelines and the experimental procedure was approved by the Stockholm Regional Ethics Committee (2019/05501).

### Stimuli and Experimental Procedure

The standard stimuli were a set of Swedish phrases with attested and unattested phonological variations in various phonological contexts: (i) coronal nasal /n/, [n] followed by labial /m/, [m] as in *utan mej* [ʉːtan mɛjː] “without me”; (ii) coronal nasal /n/, [n] followed by coronal /d/, [d] as in *utan dej* [ʉːtan dɛjː] “without you”; (iii) labial /m/, [m] followed by labial /m/, [m] as in *utom mej* [ʉːtɔm mɛjː] “except me”; and (iv) labial /m/, [m] followed by coronal [d] as in *utom dej* [ʉːtɔm dɛjː] “except you.” The deviant stimuli consisted of either attested phonological assimilations or unattested variations, created through changes from /n/ to [m] as in [ʉːtam mɛjː] and ^∗^[ʉːtam dɛjː], and through changes from /m/ to [n] as in ^∗^[ʉːtɔn mɛjː] and ^∗^[ʉːtɔn dɛjː]^4^.

A speech and language pathologist (female from Stockholm, 60 years old) produced all the stimuli in an anechoic chamber. The recordings were performed using the REAPER digital audio workstation (version 5.93; 44.1 kHz/16 bits). Acoustic analysis and manipulations were carried out on exemplars selected among several repetitions of each stimulus using Praat (version 6.0.33; Boersma and Weenink, 2014). Selected exemplars were segmented, where boundaries were determined by visual inspection of waveforms and Gaussian window broadband spectrograms (bandwidth = 260 Hz). Extracted segments were then matched for duration using the Vocal Toolkit plugin (Corretge, 2012), while preserving the other acoustic characteristics. In order to keep the deviants and standards identical and get an equal ground for the comparison, the stimuli differed from each other only in the variable segments /a/, /o/, /n/ and /m/ as well as /dej/ and /mej/. The deviant stimulus [ʉːtam mɛjː], for instance, was created out of the standard stimulus [ʉːtan mɛjː] by a splicing technique; the critical segment [n] was extracted from the relevant context and replaced with [m]. The critical segments were extracted and spliced at zero-crossings in order to avoid spurious clicks in the spliced signal, and pulses were added and deleted when necessary. To eliminate spurious clicks at the beginning and end of the stimuli, 2 ms ramps were added to the onset and offset. The length of each stimulus was 800 ms, and the divergence point between standards and deviants was at 400 ms. The acoustic quality of the stimuli was validated by independent judgment of five listeners, including the authors themselves.

The stimuli were presented in a passive auditory oddball paradigm using E-Prime (version 2.0). The stimuli were delivered via loudspeakers at a comfortable listening level while a silent movie was used to direct participants’ attention away from the auditory stimuli. The experiment had four blocks and each block consisted of 600 stimuli – 480 standards (80%) and 120 deviants (20%), following the typical probabilities of the oddball paradigm. The order of the blocks was counterbalanced across participants. The deviants were semi-randomly placed among the standards (with at least two intervening standards between the two consecutive deviants) and a random interstimulus interval (ISI) was used to avoid rhythmicity. The ISI was centered around 500 ms, with a range between 450 and 550 ms.

### EEG Data Collection and Analysis

The electroencephalography (EEG) data were collected with the BioSemi ActiveTwo system and the ActiView acquisition software (BioSemi, Netherlands) in an electrically insulated and sound-attenuated recording booth. Recordings were made from sixteen cap-mounted active electrodes (Fp1, Fp2, F3, Fz, F4, T7, C3, Cz, C4, T8, P3, Pz, P4, O1, Oz, and O2) positioned according to the International 10–20 system. A common mode sense active electrode and a driven right leg passive electrode replaced the ground electrode. Electrooculogram and nose data (used for offline referencing) were collected through external electrodes.

The EEG data analysis was performed in Matlab (version 9.4; The Math Works Inc., Natick, MA, United States) using the EEGLAB toolbox (Delorme and Makeig, 2004). The continuous EEG data were filtered using a finite impulse response band-pass filter of 0.5 to 30 Hz. The channels were re-referenced to the nose channel. The EEG data were decomposed using independent component analysis (Jung et al., 2000) and eye artifacts, which were in leading positions in the component array, were then removed from the data. On average, two components were removed. The EEG data were segmented into epochs of 1,200 ms and baseline corrected using the 100 ms pre-divergence interval. Additional artifact rejection was carried out automatically, removing any epochs containing EEG fluctuation exceeding ±100 μV (4.3% excluded trials in total).

A time window of 50 ms, centered at the peak latency, was used for the quantification of the MMN amplitude. The statistical analysis of these data was performed in SPSS (International Business Machines Corp., Armonk, NY, United States). Mean amplitude values from frontal electrodes (F3, Fz, and F4) at three time windows (120–180, 250–300, and 400–450 ms from the divergence point) were selected for the analysis. The time windows were chosen to optimally capture ERP modulations related to target phonemes or syllables in grand-average waveforms. In order to test whether MMN responses significantly differed from zero, deviant-minus-standard difference amplitudes were tested against zero with one-sample *t-*tests. To evaluate the overall effect of deviations on the ERP responses, two-way repeated-measures ANOVAs with factors Block (I–IV) and Stimuli (Standard and Deviant) were subsequently carried out in each time window. Effect sizes are given in partial η2 measures, and mean values are reported with standard deviations.

## Results

**FIGURE 1:**
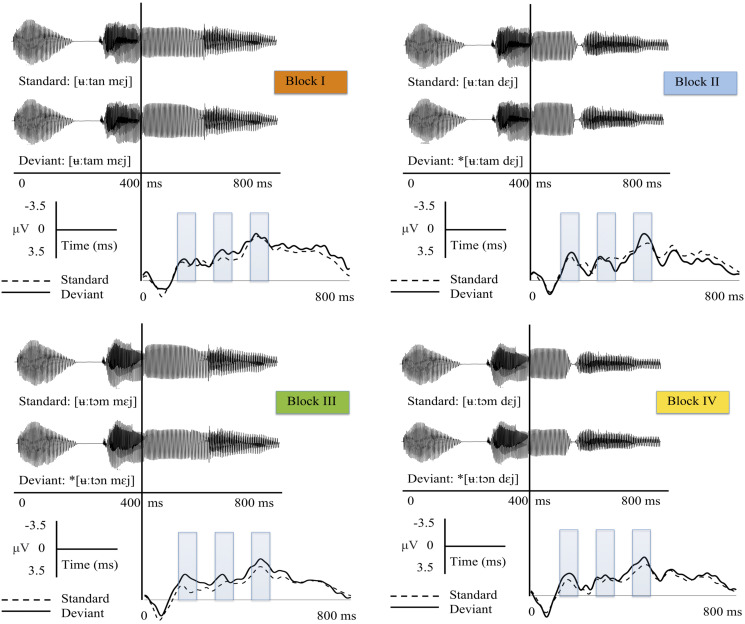
Sound and grand-average ERP waveforms (from Fz) for the standard and deviant stimuli in each block. Blocks are color-coded in line with the bar graphs presented in Figure 2. The black lines show the ERPs for the deviant stimuli, the dashed lines the ERPs for the standard stimuli. The divergence point was used as zero point in the ERP figures given that the standards and deviants were identical up to the assimilation point. The shaded bars represent time windows selected for statistical analysis. Asterisks mark inappropriate/unattested deviant sequences.

The grand-average ERPs for the standard and deviant stimuli from Fz are displayed for each block in Figure 1. The results from *t*-tests and the ANOVAs are presented in detail below and elaborated on with regard to MMN predictions.

### Results From *t*-Tests

**FIGURE 2:**
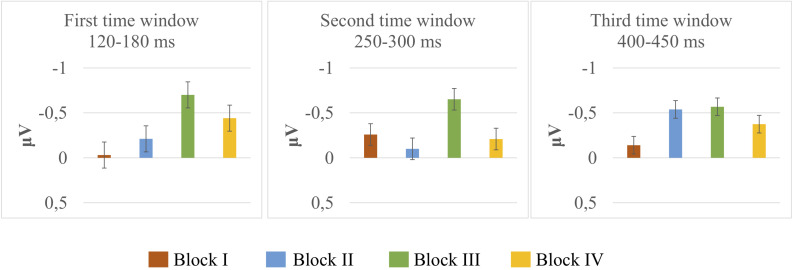
Mean and the standard error of the mean for deviant-minus-standard subtraction amplitudes extracted from the frontal electrodes (F3, Fz, and F4) in microvolts (μV) of Block I (Orange bar), Block II (Blue bar), Block III (Green bar), and Block IV (Yellow bar) at three time windows.

Deviant-minus-standard difference amplitudes were tested against zero with one-sample *t-*tests. The results of *t*-tests are presented in Table 1, and mean and the standard error of the mean for deviant-minus-standard difference amplitudes are illustrated in Figure 2. The results in the first time window (120–180 ms) indicated that the amplitudes did not differ from zero in Block I [*t(14)* = –0.122, *p* = 0.904]; Block II [*t(14)* = –0.760, *p* = 0.460]; and Block IV [*t(14)* = –1.879, *p* = 0.081]. A significant difference was present only in Block III [*t(14)* = –2.414, *p* = 0.030, *M* = –0.70, and *SD* = 1.12]. The results in the second time window (250–300 ms) showed no significant differences in any of the blocks (Block I [*t(14)* = –1.108, *p* = 0.286]; Block II [*t(14)* = –0.594, *p* = 0.562]; Block III [*t(14)* = –1.709, *p* = 0.109]; and Block IV [*t(14)* = –0.682, *p* = 0.506]). In the third time window (400–450 ms), there was a significant difference in Block II [*t*_(14)_ = –3.447, *p* = 0.004, *M* = –0.53, and *SD* = 0.60], whereas the amplitudes did not differ from zero in Blocks I [*t(14)* = –0.590, *p* = 0.565]; III [*t(14)* = –1.503, *p* = 0.155]; and IV [*t(14)* = –1.137, *p* = 0.274].

**TABLE 1:** Results of one-sample *t* tests where the amplitudes of deviant-minus-standard subtractions were tested against zero.

			*M*	*SD*
Time window 120–180 ms	Block I	*t*(14) = –0.122, *p* = 0.904	–0.03	0.97
	Block II	*t*(14) = –0.760, *p* = 0.460	–0.21	1.07
	Block III	*t*(14) = –2.414, *p* = 0.030*	–0.70	1.12
	Block IV	*t*(14) = –1.879, *p* = 0.081	–0.44	0.90
Time window 250–300 ms	Block I	*t*(14) = –1.108, *p* = 0.286	–0.25	0.90
	Block II	*t*(14) = –0.594, *p* = 0.562	–0.09	0.65
	Block III	*t*(14) = –1.709, *p* = 0.109	–0.65	1.47
	Block IV	*t*(14) = –0.682, *p* = 0.506	–0.20	1.18
Time window 400–450 ms	Block I	*t*(14) = –0.590, *p* = 0.565	–0.14	0.92
	Block II	*t*(14) = –3.447, *p* = 0.004*	–0.53	0.60
	Block III	*t*(14) = –1.503, *p* = 0.155	–0.56	1.46
	Block IV	*t*(14) = –1.137, *p* = 0.274	–0.37	1.27

Mean amplitude values (M) are reported with standard deviations (SD). * p < 0.05.

### Results From ANOVAs

Two-way repeated-measures ANOVAs with factors Block (I–IV) and Stimuli (Standard and Deviant) were carried out in each time window. The results of two-way repeated measures ANOVA in the first time window (120–180 ms) indicated no significant main effect Block [*F*_(3_, _42)_ = 1.143, *p* = 0.343, and η*2* = 0.075]. However, a significant main effect of Stimuli [*F*_(1_, _14)_ = 5.555, *p* = 0.034, and η*2* = 0.284] was found. The analysis yielded no significant interaction between these factors [*F*_(3_, _42)_ = 1.300, *p* = 0.287, and η*2* = 0.085]. In the second time window (250–300 ms), neither the main effects Block [*F*_(3_, _42)_ = 0.162, *p* = 0.921, and η*2* = 0.011] and Stimuli [*F*_(1_, _14)_ = 4.078, *p* = 0.063, and η*2* = 0.226] nor interaction between them [*F*_(3_, _42)_ = 0.751, *p* = 0.528, and η*2* = 0.051] reached significance. Similar to the first time window, in the third time window (400–450 ms) there was no significant main effect of Block [*F*_(3_, _42)_ = 1.616, *p* = 0.199, and η*2* = 0.104] but we found a significant main effect of Stimuli [*F*_(1_, _14)_ = 6.063, *p* = 0.027, and η*2* = 0.302]. There was no significant interaction between these factors either [*F*_(3_, _42)_ = 0.516, *p* = 0.674, and η*2* = 0.036]. Significant main effects of Stimuli in the first and third time windows indicated larger negative deflections for the deviant stimuli. To validate the results from ANOVAs, LMMs with Block and Stimuli as fixed factors were carried out both unstructured and with compound symmetry structure. In all cases, the results were identical to those from ANOVAs. The unstructured LMM from the first time window indicated a significant effect of Stimuli (*p* = 0.028). Block (*p* = 0.308) and Interaction (*p* = 0.371) effects did not, however, reach significance. The results from the third time window revealed similar patterns (Block, *p* = 0.181; Stimuli, *p* = 0.022; and Interaction, *p* = 0.557). The results of the compound symmetry structure in the first time window yielded a significant effect of Stimuli (*p* = 0.009). There were, however, no significant effects of Block (*p* = 0.289) and Interaction (*p* = 0.295). Similarly, in the third time window, there was a significant effect of Stimuli (*p* = 0.017) while the effects of Block (*p* = 0.069) and Interaction (*p* = 0.792) displayed no significance.

### MMN Interpretations

The results of the ANOVAs showed no significant interactions between the stimuli and blocks, indicating that the variations are treated in the same way across the blocks. Although the ANOVAs did not show any significant interactions, the grand average waveforms and the MMN responses verified by one-sample *t*-tests suggested differences in MMN elicitation that may be influenced by the variation type. The results indicated, for instance, that an unattested change (i.e., a labial-to-coronal change in a labial context, [ʉːtɔm] > [ʉːtɔn] + [mɛjː]) elicited a significant MMN response at an early stage (Block III), and an attested change in an inappropriate context (i.e., a coronal-to-labial change followed by a coronal context, [ʉːtan] > [ʉːtam] + [dɛjː]) elicited a significant MMN response at a later time window (Block II), whereas the other variations did not elicit significant MMNs (see Figure 2 and Table 1). In the early time window, there was further a tendency for an MMN response to another unattested change (i.e., a labial-to-coronal change in a coronal context, [ʉːtɔm] > [ʉːtɔn] + [dɛjː]), yet this response was not robust enough to reach significance (Block IV).

## Discussion

Transformation of auditory input into a meaningful representation is affected by several constraints, including attested and unattested phonological variations in the speech signal. The present paper investigated the consequences of attested phonological assimilations and unattested phonological variations in lexical access, and elaborated on different theoretical accounts for phonological assimilation. The attested case of phonological variation introduced by Swedish nasal regressive place assimilation was scrutinized in appropriate and inappropriate phonological contexts. For comparison, an instance of unattested phonological variation that does not appear naturally in the language was investigated in the relevant contexts. The results showed no significant interactions between the variations, indicating that the correct forms were retrieved from the signal, irrespective of the variations. However, there were differences in MMN elicitation that may be influenced by the nature of variations and phonological contexts. In the rest of the paper, we discuss these findings in light of various theoretical accounts for phonological assimilation and their MMN predictions as presented in the Introduction section (see Table 2).

**TABLE 2:** Excerpts from each block and the relevant theoretical accounts and their MMN predictions.

Block	Change	Context	Standard	Deviant
Block I	Coronal [n] > Labial [m] Attested	Labial [m] Appropriate	[ʉːtan mɛjː]	[ʉːtam mɛjː]
Block II	Coronal [n] > Labial [m] Attested	Coronal [d] Inappropriate	[ʉːtan dɛjː]	[ʉːtam dɛjː]
Block III	Labial [m] > Coronal [n] Unattested	Labial [m]	[ʉːtɔm mɛjː]	[ʉːtɔn mɛjː]
Block IV	Labial [m] > Coronal [n] Unattested	Coronal [d]	[ʉːtɔm dɛjː]	[ʉːtɔn dɛjː]
**Theoretical account**	**MMN predictions**			
Top-down lexical compensation	No difference across Blocks			
Feature underspecification (FUL)	Smaller MMN for Blocks I and II in comparison to Blocks III – IV			
Regressive inference	Smaller MMN for Block I in comparison to Blocks II – III – IV			
Feature parsing	Smaller MMN for Blocks I and IV in comparison to Blocks II – III			

*Block I: an attested change in an appropriate context – a coronal-to-labial change followed by a labial context; Block II: an attested change in an inappropriate context – a coronal-to-labial change followed by a coronal context; Block III: an unattested change – a labial-to-coronal change followed by a labial context; and Block IV: an unattested change – a labial-to-coronal change followed by a coronal context.*

According to the simple top-down lexical compensation account (e.g., Marslen-Wilson and Welsh, 1978; Samuel, 2001; Bölte and Coenen, 2002; Darcy et al., 2009; see also the tolerance-to-mismatch approach in Gow, 2001), no MMN difference is expected across the experimental blocks since the deviation from the canonical form will be compensated for at a lexical level, using semantic cues, irrespective of the nature of variation and the following phonological context. Given that there was no significant interaction between any of the deviations and the phonological context, the results are claimed to be in line with this lexical compensation account. It can be argued that the attested and unattested changes were treated in the same way across the appropriate and inappropriate contexts, and the variations did not incur an apparent cost in lexical access. The listeners may have successfully repaired the deviations since the extracted inputs from the deviants differed from the lexical representations formed by the standards only in one feature. Given also that this difference occurred at the end of the words, which underwent a phonological change, and that the difference between/m/and/n/is subtle, the brain might have corrected and compensated for the differences between these forms after several repetitions. These results are in line with the theories of spoken word recognition that assume a top-down influence of lexical representations on the activation of smaller perceptual units rather than a fully bottom-up flow of information (Marslen-Wilson and Welsh, 1978; Samuel, 2001; Bölte and Coenen, 2002; Darcy et al., 2009).

In contrast to the previous MMN studies, which argue for assimilatory processes operating on a pre-lexical level and argue in favor of the feature parsing and inference accounts (Mitterer and Blomert, 2003; Mitterer et al., 2006; Tavabi et al., 2009), the present paper indicated that the attested phonological assimilations as well as unattested phonological variations are compensated for at the lexical level. Although the current results do not provide unequivocal support for the other accounts reviewed in the present paper, they should not be dismissed fully. The MMN responses verified by one-sample *t*-tests suggested differences in MMN elicitation that may be affected by the nature of variation and the phonological context. A late MMN response to Block II (an attested change in an inappropriate context) and an early MMN response to Block III (an unattested change in the labial context), are partially in line with the MMN predictions of the FUL, regressive inference and feature parsing accounts, which are further discussed below.

The FUL account (e.g., Lahiri and Marslen-Wilson, 1991; Lahiri and Reetz, 2002, 2010) predicts different MMN responses to [ʉːtan] > [ʉːtam] and [ʉːtɔm] > [ʉːtɔn], yet regardless of the following phonological contexts, [mɛjː] and [dɛjː]. According to this account, a smaller MMN response is predicted to the deviants in both Block I and II, since the deviants – a change from [n] to [m] as in [ʉːtan] > [ʉːtam] – will be tolerated given the underspecification of coronal/n/and therefore a no-mismatch condition. The significant MMN response in Block II thus contradicts the FUL account. The MMN response in Block III is, however, in line with the FUL account, which predicts a clear MMN response to the deviants in Blocks III and IV, since the deviants – a change from [m] in [ʉːtɔm] to [n] as in [ʉːtɔm] > [ʉːtɔn] – will not be tolerated given that nasal assimilation only applies to the coronal nasal and a change from [m] to [n] creates a mismatch condition. However, according to the FUL account, this MMN response should be present in both phonological contexts, yet the response was not robust enough to reach significance in the coronal context (see Block IV).

Given the early MMN response in Block III and the marginally significant early MMN response in Block IV, one can still argue that the labial-to-coronal change was, in fact, directly perceived as incorrect prior to the following context, providing support for the FUL account. One can, however, also argue that the MMN response in Block II was late likely because the coronal-to-labial change remained acceptable until the onset of the following context; [ʉːtam] was perceived as incorrect only after encountering the [dɛjː] context, which, in turn, provides evidence for the regressive inference account.

In contrast to the FUL account, the regressive inference account argues that assimilatory changes are processed faster and more accurately in phonologically appropriate contexts (e.g., Gaskell and Marslen-Wilson, 1996, 2001; Coenen et al., 2001; Mitterer and Blomert, 2003; Mitterer et al., 2006; Gaskell and Snoeren, 2008; Tavabi et al., 2009). The regressive inference account predicts different MMN responses to [ʉːtan] > [ʉːtam] depending on the following context, [mɛjː] and [dɛjː]. In Block I, the deviant will be tolerated by assimilation of the [n] in [ʉːtan] to [m] due to the following phonological context [mɛjː], and accordingly no MMN response is predicted to the deviant. In Block II, on the other hand, the deviant will not arise by assimilation of the /n/ in [ʉːtan] to [m], due to the lack of a following context for assimilation, and an MMN response is predicted to be elicited to the deviant. The MMN response in Block II therefore provides support for the regressive inference account. This finding is also in line with previous research, which has indicated larger MMN response to an inappropriate context for assimilation (Mitterer and Blomert, 2003; Mitterer et al., 2006).

The reported late MMN response to Block II and early MMN response to Block III provides support for the feature parsing account, which predicted an attenuated MMN response to the deviants in Blocks I and IV compared to the deviants in Blocks II and III. The feature parsing account argues that an assimilated segment accomodates information not only about the original place of articulation present in the signal but also about the following segment (Gow, 2003). In this account, as long as they follow the grouping principles, no difference is expected between an attested phonological assimilation and an unattested phonological variation (see the priming experiment in Gow, 2001). The current MMN findings do not provide direct evidence for the feature parsing account since the unmodified coronals and labials were used as assimilated segments rather than intermediate, phonetically ambiguous segments, as used in Gow, 2003. However, given that the labiality of the assimilated segment in [ʉːtam] will be associated with the labiality of the following context in [mɛjː], leaving only the coronal property to be associated with the final segment of the word candidate as in [ʉːtan], and the coronality of the assimilated segment in [ʉːtɔn] will group with the coronality of the following segment in [dɛjː], leaving thus only the labial property to be related to the final segment of the word candidate in [ʉːtɔm], smooth word recognition was possible in variations as in Blocks I and IV, and accordingly smaller MMN responses were elicited to these variations.

The present pattern of results is in line with previous research, which argues that general perceptual preferences and phonetic details have an impact on the kind of assimilation rules applied (e.g., Mitterer et al., 2006). For instance, an indifference to contextual appropriateness, reported for the second experiment in Mitterer et al. (2006), was shown to depend on the acoustic details of the stimuli; the authors could in fact document the impact of context on assimilatory processes after changing the phonetic details of the stimuli (see for instance the fifth experiment). One can also argue that the current results indicate that the consonant sequences might favor the same place of articulation; if the change leads to a mismatch between the assimilated segment and the following segment with regard to the place of articulation, a larger MMN response is elicited, indicating a low-level perceptual processing independent of the nature of variation.

To conclude, the processing of phonological variations is contributed by lexical representations. For successful lexical access, there is no need for a close match between the auditory information extracted from the signal and lexical representations. Even unattested phonological variations successfully activate lexical representations, and a minimal mismatch between the features that are extracted from the signal and the features that comprise the lexical representations is compensated for at the lexical level. The results, however, indicate a hint of pre-lexical processing and point out context sensitivity to some extent in a similar fashion suggested in the feature parsing account. These findings thus raise the need for further comparisons, which can be obtained by changing the nature of the stimuli by introducing gradient modification of place of articulation, and by testing other target languages. By establishing the neural correlates of attested and unattested phonological variations and their consequences in lexical processing, the present study contributes to the understanding of inherently variable spoken language communication and automatic lexical access, which is particularly important given the rapid nature of spoken communication. The findings are relevant for explaining our ability to effectively recognize words despite variations as a result of assimilatory process as well as variations introduced by other factors such as speech rate, dialect and background noise. Most importantly, the present study attempts to provide a unified account of spoken language processing by deriving and testing the predictions of competing theoretical accounts on assimilatory processes. Revealing not only low-level perceptual processing of phonological units, but also higher-level lexical processing, the present pattern of results harmonizes the bottom-up and top-down theories of speech processing.

## Data Availability Statement

The raw data supporting the conclusions of this article will be made available by the authors, without undue reservation.

## Ethics Statement

The studies involving human participants were reviewed and approved by Stockholm Regional Ethics Committee (2019/05501). The patients/participants provided their written informed consent to participate in this study.

## Author Contributions

HZ, TR, SY, and VC: conception and design of the work. HZ: experimental work and drafting the manuscript. HZ, TR, SY, and VC: revision and final approval of the version to be published. All authors contributed to the article and approved the submitted version.

## Conflict of Interest

The authors declare that the research was conducted in the absence of any commercial or financial relationships that could be construed as a potential conflict of interest.

## Footnotes

1 Delative and adessive suffixes are Hungarian case marking suffixes, representing “from” and “at,” respectively.

2 It should, however, be noted that the labial nasal assimilates to a labiodental before a labiodental fricative as in *dom farorna* “those dangers” [dɔɱ fɑːrʊɳa].

3 The coronal and labial properties are associated with the final segments of the preceding words depending on the semantic context.

4 The normative spellings of these pronouns are <mig> and <dig>.
